# Research highlights for issue 6: the applicability of model system research

**DOI:** 10.1111/eva.12182

**Published:** 2014-06-27

**Authors:** Britt Koskella

Our ability to apply evolutionary theory is necessarily limited by our understanding of natural systems. Unfortunately, given the finite amount of researcher time and funding, we are faced with a trade-off between studying many systems shallowly and studying fewer systems in more depth. The handful of ‘model systems’ that have emerged thus far act as workhorses across disciplines, allowing for a more complete understanding of each system in fields from molecular genetics and evolution, to development, to physiology, to whole organism biology and ecology. However, given how few such model systems exist, it remains to determine how generalizable they are to less well-studied species and the natural world as a whole.

Among the model systems offering a wealth of insight across fields are the yeast *Saccharomyces cerevisiae*, the nematode *Caenorhabditis elegans*, the fly *Drosophila melanogaster*, and the plant *Arabidopsis thaliana*. These systems, among others, became models due in part to their ease of use in the lab, relative ubiquity, short generation time, and, in part, due to chance. The breadth of knowledge gained from the study of these and other model systems has driven progress across fields and has allowed for multidisciplinary research and cross talk among researchers that may not have otherwise come together. More recently, there has been a renewed interest in examining these systems back in the field and expanding the work to closely related species to determine whether our knowledge from the laboratory is broadly applicable in nature.

In terms of real life application, the model yeast *Saccharomyces cerevisiae* has the clearest relevance given its use in fermentation processes, the evolutionary history of which has been recently reviewed by Dashko et al. (2014). However, the tremendous toolbox of genetic techniques that is available has also made *S. cerevisiae* a central player in answering more basic questions in biology. Recent work by Serero et al. (2014) used a range of molecular approaches to examine mutation accumulation of both wild type and mutator strains of *S. cerevisiae* (i.e., strains with deficiencies in so-called ‘genome maintenance’ genes). By comparing the genome-wide mutational landscape across mutator types, they demonstrate strain-specific and complex effects of mutagenesis on chromosomal structure, mutations, and aneuploidy. The study emphasizes the tremendous diversity of the mutational landscape and provides an approach for examining genomic variation during clonal evolution, as occurs for example during tumor development in cancer.

The nematode *Caenorhabditis elegans* has acted as powerful model system for studying evolution due in part to the ability of researchers to freeze and resurrect individuals. A recent review by Gray and Cutter (2014) highlights the great potential the system still holds in exploring mutational processes, mating system and life-history evolution, and host-pathogen coevolution. For example, a new study by Sikkink et al. (2014) has examined the importance of phenotypic plasticity in adapting to extreme environments using the outcrossing sister species *C. remanei*. They experimentally selected for worms that were able to better withstand heat shock during development and found both increased tolerance of heat shock and altered phenotypic plasticity when reared in a novel environment, emphasizing both the role evolution can play in shaping plasticity and the importance of plasticity in allowing adaptation to novel and/or extreme environments.

Research on the fruitfly, *Drosophila melanogaster*, has paved the way for our understanding of genetics but has also been central to addressing questions regarding the impact of symbionts and pathogens on eukaryotic fitness (reviewed in Fauvarque 2014). Recent work by Versace et al. (2014) experimentally evolved populations of *D. melanogaster* infected with *Wolbachia* from multiple clades under hot or cold environments for 37 generations to test for changes in symbiont composition across environments. They discovered rapid increase in infection rates across replicate populations and treatments, suggesting a strong fitness advantage to hosts carrying the symbiont, as well as striking shifts in the composition of the *Wolbachia* community under cold, but not hot, environmental conditions. Studies of *Drosophila* have also been useful in understanding pest emergence and evolution. For example, a study by Atallah et al. (2014) has compared those *Drosophila* species that feed primarily on rotting fruit with those species that have switched to live fruit. Through morphological comparisons of the pest *D. suzukii* with its close relatives, the authors propose an evolutionary model to explain the modification of the ovipositor that allows puncture of susceptible fruits, demonstrating the utility of an evolutionary framework for addressing questions of pest emergence and management.

Finally, research focused on *Arabidopsis thaliana* plants has offered key insights to the genetics of plant adaptation, plant development, and plant–pathogen interactions. Two recent studies have examined natural populations of *Arabidopsis* to better understand the plant's ability to move beyond current range limits. First, Wolfe and Tonsor (2014) took advantage of a natural elevational gradient of temperature and precipitation to examine plant adaptation to increase heat and drought. By exposing 48 lineages from across the natural gradient to increasing temperature and decreasing precipitation, they show that 10 of the 12 traits measured differ across the elevations and that populations from the low elevations are most fit in the face of increased heat and drought. In particular, these plants have faster bolting and earlier fruit ripening than those from high elevations, suggesting adaptation toward avoidance of spring heat and drought. In another study, Griffin and Willi (2014) examined natural populations of another *Arabidopsis* species, *A. lyrata*, across North America to test whether self-fertilization is more common at the edge of the species range, where effective population size is likely to be lower. Using population surveys and microsatellite markers, they were able to demonstrate at least seven independent transitions from outcrossing to selfing, all of which occurred at the edge of the species range where diversity was lower. Understanding such evolutionary shifts toward self-fertilization offers important insight to the ability of a species to expand its range and potentially to become invasive.

These recent studies highlight the great potential model systems hold for applying evolutionary theory in large part due to their amenability to laboratory conditions and genetic/genomic tools. Overall, it is clear that the knowledge gained from the study of model systems has been greater than the sum of its parts, but the generalizability of such knowledge to nonmodel systems, especially when it comes to translational research, remains an open question.
